# The syndrome of inappropriate antidiuresis after vaccination against COVID-19: case report

**DOI:** 10.1186/s12879-021-06690-8

**Published:** 2021-09-25

**Authors:** Gregor Lindner, Basil Ryser

**Affiliations:** Department of Internal and Emergency Medicine, Buergerspital Solothurn, Schoengruenstrasse 42, 4500 Solothurn, Switzerland

**Keywords:** SIADH, Hyponatremia, COVID-19 vaccination, Case report

## Abstract

**Background:**

The Syndrome of Inappropriate Antidiuresis (SIADH) has been described to be associated with a multitude of conditions and medications, including the severe acute respiratory syndrome coronavirus 2. We describe the case of a patient with newly diagnosed and symptomatic SIADH after receiving the second COVID-19 vaccination not explained otherwise.

**Case presentation:**

A 79-year-old male person was admitted to the emergency department due to a worsening of his general health state expressed by weakness, fatigue and anorexia. Vital signs and clinical findings were normal, in particular the patient was considered to be euvolemic. Laboratory investigations revealed a serum sodium of 117 mmol/L, a serum osmolality of 241 mosm/kg and a urea of 1.2 mmol/L with creatinine within normal range. Urine chemistry showed a urine osmolality of 412 mosm/kg and urine sodium of 110 mmol/L. TSH, C-reactive protein, and basal cortisol levels were normal. Under therapy with balanced crystalloid fluids, hyponatremia worsened and in absence of diuretic medications, diagnosis of SIADH was made. Since fluid restriction was not sufficiently effective, oral urea was administered. Under this therapy regimen hyponatremia resolved.

**Conclusions:**

Local as well as systemic reactions have been described for the new mRNA-based vaccines including pain and fever. Therefore, it is imaginable that the vaccine might trigger SIADH in some patients.

## Background

The Syndrome of Inappropriate Antidiuresis (SIADH) is characterized by a low serum sodium, reduced serum osmolality, relatively high urine osmolality and urine sodium together with a clinically euvolemic patient in absence of diuretic medication [1]. SIADH has been described to be associated with a multitude of conditions and medications with diseases of the lung being quite prominent [1]. Only recently, SIADH was also described in patients infected with the Severe Acute Respiratory Syndrome Coronavirus 2 (SARS-CoV-2) [2, 3]. To this date, intensive vaccination programs have started world-wide in order to fight the pandemic [4]. In our country at this time, only Spikevax (mRNA-1273, Moderna) and Comirnaty (BNT162b2, Pfizer-BioNtech) were approved by our local drug administration. They both contain messenger ribonucleic acid (mRNA) coding for spike-proteins of the SARS-CoV-2 virus. The mRNA is transported into hosting cells in lipid nanoparticles, where the proteins are synthesized and presented on the cell membrane [5, 6]. This leads to an immune reaction against the presented antigens, which results in a T- and B-Cell activation and consecutively in a humoral and cellular immune response [5, 6]. The most common side effect is a solicited local reaction [5, 6]. Furthermore, common side effects reach from fever, headache and fatigue to lymphadenopathy, myalgia, arthralgia, nausea/vomiting and chills. Unfortunately, there are also rare cases of anaphylactic shock after receiving either of the mentioned vaccines. Recently, a case series of Myocarditis and Pericarditis associated with COVID-19 vaccination was published [7]. Since vaccination programs started, a lot of case reports were published describing a multitude of pathologies possibly related to the vaccination. They are reaching from thyreoiditis, over Guillan-Barré syndrome, to stroke-mimics and uveitis, to only mention a few [8–11]. We describe the first case of a patient with newly diagnosed and symptomatic SIADH after receiving the second COVID-19 vaccination not explained otherwise.

## Case presentation

A 79-year-old male person was admitted to the emergency department on March 06th 2021 due to a worsening of his general health state expressed by weakness, fatigue and anorexia. The patients' previous medical history was remarkable mainly for an ischemic cerebrovascular insult in 2019, which probably occurred on basis of microangiopathic disease. Moreover, gastritis and gastroesophageal reflux disease was known. The patient was on a regular medication of clopidogrel 75 mg as well as pantoprazole 40 mg once daily, respectively. non-steroidal anti-inflammatory drug (NSAID) use was denied.

Clinical examination on admission showed a patient in slightly reduced general state of health, normotensive blood pressure values, a heart rate of 66/min, a peripheral oxygen saturation of 97% on ambient air as well as a temperature of 37 °C. The patient did not have any peripheral edema and was considered to be euvolemic.

Laboratory investigation revealed a serum sodium of 117 mmol/L on admission, potassium of 4.3 mmol/L and a serum osmolality of 241 mosm/kg with a serum creatinine of 67 µmol/L and a urea of 1.2 mmol/L. TSH and C-reactive protein levels were normal. Hyponatremia was confirmed in arterial blood gas analysis, to exclude pseudo-hyponatremia. The patient was administered intravenous crystalloid solutions and admitted to the medical ward. In order to determine the etiology of hyponatremia a urine chemistry was ordered: Urine osmolality was 412 mosm/kg and urine sodium 110 mmol/L. In order to rule out adrenal insufficiency a basal cortisol was ordered for the next day, which was in the normal range (101 nmol/l). On admission laboratory results are given in Table 1. The patients' serum sodium further declined to 116 mmol/L with the intravenous crystalloid solution. Moreover, respiratory multiplex PCR (Influenza A, Influenza B, SARS-CoV2, RSV A and B) was negative.

**Table 1:** Serum and urine chemistry results on admission

Parameter	Reference range	Case
Sodium	136–145 mmol/L	117
Potassium	3.6–5.1 mmol/L	4.3
Calcium	2.20–2.60 mmol/L	2.20
Phosphate	0.74–1.52 mmol/L	0.93
Osmolality	270–295 mosm/kg	241
Glucose	4.6–6.1 mmol/L	4.8
Creatinine	49–90 µmol/L	67
Urea	3.5–7.2 mmol/L	1.2
C-reactive protein	< 5.1 mg/L	3.3
TSH	0.35–4.94 mU/L	3.19
Basal Cortisol	101–536 nmol/L	101
Urine		
Sodium	mmol/L	110
Creatinine	µmol/L	9.592
Osmolality	mosm/kg	412

On basis of a clinically euvolemic patient, absence of diuretic medication, high urine osmolality and urine sodium of 110 mmol/L together with a relevant hypoosmolar hyponatremia the diagnosis of SIADH was made. Fluid restriction at 1.000 ml/day was started, which led to a slight increase in serum sodium. In order to assess for a triggering factor for the SIADH a detailed history was obtained excluding any medications taken in addition to the previously mentioned ones. Moreover, computed tomography of the chest was performed since the patient was a former smoker. However, no signs for malignancy, inflammation or infection were found. Pantoprazole was taken already for a long time and clopidogrel is not known to be associated with SIADH. Taken together, it was supposed that the potential trigger for the SIADH might be the second vaccination against COVID-19 (mRNA-1273, Moderna) the patient received on February 26th 2021 (the first vaccination was injected 14 days earlier).

Since fluid restriction was only moderately effective, oral urea was administered first at 30 g/day and later at 45 g/day. Under this therapeutic regimen, the serum sodium concentration increased to 133 mmol/L on March 12th 2021. The patient always reported to tolerate interventions very well, in particular also the bitter taste of urea. The initially expressed weakness and fatigue improved markedly and the patient was discharged the same day with appointments in order to perform a urea-free interval in 7–14 days post-discharge for evaluation of potential resolving of SIADH. March 17th a control of serum sodium yielded a value of 139 mmol/L and urea therapy was paused in order to assess for resolvement of SIADH.

## Discussion and conclusions

We are reporting the first case of SIADH in the context of previous vaccination against COVID-19 in a 79-year-old patient, who presented with marked hyponatremia.

Hyponatremia has been described to be a common occurrence in various lung diseases and is associated with adverse outcome of patients [12]. Hyponatremia in the context of SIADH has also been linked to several conditions of the lungs including COVID-19 [1–3]. During the last months vaccination programs against COVID-19 started internationally, administering immunizations to an increasing number of people. Local as well as systemic reactions have been described for the new mRNA based vaccines including pain, local erythema, headache, fatigue and fevers [13]. Therefore, it is imaginable that the vaccine per se might trigger SIADH in some patients, since SARS-CoV-2 infections are also associated with SIADH [2]. However, pathophysiology leading to SIADH remains unclear. In the present case, no alternative triggering agent could be identified, leaving the previous vaccination as a plausible cause. The main limitation of this case report is that causality of SIADH can only be presumed but not proofed. Further research is necessary to show if there is an actual correlation between mRNA SARS-CoV-2 vaccinations and SIADH, and to understand the underlying pathophysiology. Awareness of the potential side effect is crucial in physicians caring for patients suffering from prolonged symptoms after COVID-19 vaccination in order to identify and treat these patients. In conclusion, when hyponatremia is occurring after mRNA COVID-19 vaccination, treating physicians should be aware of possible SIADH.

## Data Availability

Data sharing is not applicable to this article as no datasets were generated or analyzed during the current case report.

## Abbreviations

SIADH: Syndrome of Inappropriate Antidiuresis
SARS-CoV-2: Severe Acute Respiratory Syndrome Coronavirus 2
NSAID: Non-steroidal anti-inflammatory drugs
mRNA: Messenger ribonucleic acid
